# Supplementary material for: Intimate partner violence experience, support seeking and coping strategies among pregnant women in Southwestern Uganda
Source: PLOS Glob Public Health. 2026 Jan 5;6(1):e0002606. doi: 10.1371/journal.pgph.0002606 (PMC12768344; doi:10.1371/journal.pgph.0002606)
Supplement: S1 Table — (DOCX) [file pgph.0002606.s001.docx]

## Interview guide (English version)

| **Ser. no** | **Socio-demographic characteristics and obstetric history of participants** | | |
| --- | --- | --- | --- |
| **1.** | Age in completed years | | |
| **4.** | **Occupation**  1=Unemployed 2=Employed/salaried  3= self-employed/business | **5.** | **Religion**  1 = Christian 2 = Moslem  3 = (others specify) |
| **6.** | **Marital status**  1 = Married 2 = single 3 = separated  4 = Others (specify) | **7.** | Number of pregnancies |
| **Women’s experiences with IPV**  ***I understand that you have experienced IPV before, kindly tell me about your current experience (s) until there is nothing to tell me about. There is no wrong or right answer.***  ***Probes***  ***IPV experience***   1. Kindly describe your experience with intimate partner violence (IPV) in the current pregnancy. 2. How did you feel when you experienced IPV? 3. Describe the various ways you have been met with violence during this current pregnancy. 4. Kindly explain what you think drives or brings about IPV experience during pregnancy (at home and in the community).   ***Support-seeking and coping strategies***   1. Kindly tell me more about what you did when you experienced IPV? 2. Describe how you sought support after an IPV experience. 3. Tell me about what may facilitate or hinder one from obtaining support following an IPV experience. 4. Kindly describe how you coped with the IPV situation? (psychologically, financially, etc) 5. Describe your experiences with health care providers regarding discussions about IPV during your current pregnancy.   **Thank you very much for your time!** | | | |
