# Supplementary material for: Intimate partner violence experience, support seeking and coping strategies among pregnant women in Southwestern Uganda
Source: PLOS Glob Public Health. 2026 Jan 5;6(1):e0002606. doi: 10.1371/journal.pgph.0002606 (PMC12768344; doi:10.1371/journal.pgph.0002606)
Supplement: S2 Table — (DOCX) [file pgph.0002606.s002.docx]

**S2 Table: Themes that were identified from participants' responses**

| **Theme** | **Subtheme** | **Code** | **Quotes** |
| --- | --- | --- | --- |
| Power and control dynamics in intimate relationships | Physical violence | - Stepping on a pregnant belly - Being assaulted physically during pregnancy - Using physical force to assert dominance | *“……he recently stepped on my pregnant belly." (Phiona)*  *“He almost choked me to death, but I escaped and never went back.” (Ronah)*  *"My husband beats me and always leaves bruises."*(Betty)  “My husband slaps me on the face, ears, and head. I feel so bad and I’m always furious.**”** (Marion) |
|  | Reproductive violence | - Being accused of failure to conceive - Being abandoned due to pregnancy disclosure - Abuser acting strangely after conception. | *“Things were fine until I told him I was pregnant, and then he started acting weird….”* (Annette)  *"I don't know where he is now; he left me when I told him I was pregnant and blocked my phone number."* (Peace)  *“He accused me of not wanting to get pregnant and hit me on the forehead, I had even conceived around that time but both of us hadn’t realized.”* (Allen) |
|  | Psychological violence | - Being verbally abused by my partner - Being accused of extramarital affairs - Feeling jealous when I speak to other men - **Chasing me out when the baby cries** | *“Every time he tells me that I have other men, yet I don’t. This makes me feel bad.”* (Mable)  *“He's jealous when I talk to other men and wants to know what we said.”* (Irene)  *“He abuses me so much, I just feel so empty inside, like there is a big hole that can’t be filled.”(Juliet)*  *“It's hurtful that he chases me out of the house late at night when the baby cries, like it's my fault.”* ( Rehema ) |
|  | Sexual violence | - Being forced to have sexual intercourse - Being forced to have sex in uncomfortable positions - Non-consensual sexual intercourse - Disregarding the woman’s pain and discomfort - Expecting sexual availability regardless of the condition - Forcing sex immediately after laborious activities | *"My husband forces me to have sex even when I'm not interested or feeling it."(Doreen)*  *“Laughs….my husband wants to be on top of me during sexual intercourse yet am pregnant and I feel a lot of pain, I have told him that it’s not comfortable, but he does not listen he does it every day and it hurts me.”* (Rose)  *“My husband has bad behaviors, I come from the garden very tired, and he forces me to have intercourse with him there and then, yet he spends the whole day in bed.”* (Allen ) |
|  | Financial Control and Manipulation | - Being prevented from working for pay - Being denied funds to seek medical attention - Refusing to buy food for the family - Not being involved in the purchasing of family assets. - Financial secrecy and exclusion from decision-making - Using economic dependency as a means of control | *"I'm educated, but he's keeping me home he doesn’t want me to get a job and earn money.”* (Juliet)  *"I started this salon alone, but he takes all my earnings. He's always around when I talk to customers."* (Rose )  *“I’m a housewife but when I’m sick he cannot give me money to go for treatment like he doesn’t care and it hurts.” (Scovia)*  *"He's buying property behind my back. I think it's because I'm not financially contributing, but I'm worried he might be planning to marry another woman."(Joyce)*  *“He works but always leaves no money and food at home, yet he wants to eat when he comes back.”* (Janet) |
| Drivers of IPV | Intergenerational violence | - Witnessing violence in the family of origin - Citing their mother-in-law’s similar abuse | *“What is happening to me occurs everywhere I used to see this during my childhood…. My Dad Hmmm.” (Generous)*  *“This is not new to me because I used to witness my father beating my mother and occasionally kicking her out of the house." (*Brenda*)*  *"Considering the way my spouse's father has treated her in the past, my mother-in-law told me to be patient and know that I will succeed in the end."*  ( Rehema ) |
|  | Unequal power dynamics in the household | - Men being the sole decision – maker - Dismissing the woman’s input in a home - Minimizing the woman’s input | *“In our home, he is the one who decides, and he usually reminds me about it, if I go against it he will beat me. When I search for advice many people tell me, yes, it’s a man to make decisions in a home…. I disagree, and I’m labeled big-headed.”* (Medius)  *“My husband is trying to control me by buying everything for our home and he destroys what I buy. He wants me to feel dependent on him which makes me feel bad."* (Becky*)*  *“When I buy an item at home, he lies to children that he gave me the money, just to show them that I’m useless and that everything belongs to him.”* (Kellen) |
|  | Differences in traditional gender roles and social norms | - Expecting women to do the hard work - Perceiving men to be superior - Feeling respected - Encouraged to endure the abuse | *“My husband asserts that since he pays the school fees and constructed the house, I should undertake the donkey work, even when I am unwell, he refuses to assist me.”(*Rita*)*  *"I'm pregnant, but my husband still expects me to do all the hard work. I refused to carry a heavy load of bananas, and he hit me as a punishment."* (Rose)  *"Despite the challenges I go through, I like how people respect me because I'm married, nobody will give you respect if you have no husband."* (*Sarah)*  *My mother-in law always tells me to endure like women of long ago such that I build the home and continue taking care of my children (*Irene*)*  *"He can slap me if I argue with him and I can’t compete with a man so I just let him be and I remain with my problems."* (Sonia) |
| Coping strategies | Active coping strategies | - **Seeking support from** friends, family, and pastors - Praying to God - Thinking positively - Reporting to authorities | *“Almost every day I call my mother such that we talk about it and I relieve the stress off me, my mother tells me to keep quiet and ignore him.”* (Sandra)  *“I usually talk to my sister who counsels me and tells me to be strong and patient and indeed I’m strong and managing.”* (Rita)  *“I always pray to God and my pastor (Church leader) prays for me*; *I have faith that he'll eventually change.”* ( Sonia)  *“……After injuring me I reported to the chairman and police, he was counseled and now he better.”* (Allen)  *"I try to think positive by focusing on my friends which helps me to forget."* (Brenda) |
|  | Accommodating abuse | - Agreeing to everything their partner said - **Some kept quiet** - State of Acceptance - **Tolerated partner** - Understanding spouse’s preferences | *“Whatever he says whether wrong or right I say yes yes…to avoid being beaten."* (Marion)  *“Most of the time he quarrels, and I pretend to be a fool, so I always keep quiet to avoid slaps and punches.”* (Rita)  *“Even if I argue he will not value my opinion, so I just keep quiet and listen, that is the only medicine.”* (Betty)  *“…. everyone has their battles; I choose to accept my husband despite his shortcomings. Many families are in a similar dilemma.”* (Rehema)  *"I've succeeded by making an effort to learn about his likes and dislikes, now what else would I do?"* (Joyce) |
| Barriers to Seeking Support | lack of awareness | - unawareness of the midwife’s role in IPV management - Being unaware of how to approach midwives | *“I don't think a midwife has a role to play in this abuse, is she, my aunt?”* (Irene)  *"I want to tell the midwife about my problems at home, but I don't know how to start."* (Rose) |
|  | Negative experiences with health care providers | - Failing to inquire about IPV. - Midwives not focusing beyond abdominal examinations - Understaffing. - midwives lack sufficient time - Failing to receive help after IPV disclosure | *"You're the first healthcare provider to ask about the abuse I suffer at home."* (Betty)  *"It seems like family problems aren't their concern."* (Sarah)  *“I usually see one midwife caring for us and yet the line is very long….”* (Irene)  *“………. Midwives have their problems! then I also add my own.”* (Becky)  *“The midwives have limited time for us during prenatal care……….”* (Kellen)  *"I told my husband's friend about the abuse, but he never helped. I don’t see the reason for seeking more help."* (Sonia) |
|  | Partner dependence | - Still having sexual intercourse with the perpetrator - Perpetrator providing for the family financially | *"I tell myself I'm lucky because he provides for us, even though he's mean."* (Marion)  *"If he is still interested in having sexual relations with me and pays fees for the children, it shows he still loves me so I cannot think of separating from him. I will bear his abuses.”* (Generous) |
|  | Shame and guilt | - Hiding the reason for ongoing injuries - Suffered abuse twice in different relationships and feared judgment. - Fearing to be laughed at. - Seeing IPV as a private family matter - Desiring to preserve the abuser’s reputation. | “*I lied to healthcare workers about the injuries I sustained during the fight. I felt it was embarrassing to disclose."* (Janet)  *"I don't tell anyone because they'll spread the rumors that I was beaten."* (Generous)  *"I'm sticking in this relationship despite its problems because I've been hurt before. If I go, I'm worried about what people would say."* (Sandra)  *“Those are bedroom issues that shouldn’t be discussed outside and if you tell any will go telling others about what happened and that is humiliating”* (Annette)  *“I keep those issues to myself because whoever I tell will laugh at me”* (Doreen)  *"I keep our home life a secret to protect his public reputation."* (Kellen) |
|  | Fear | .   - Threatening to abandon me - Fearing retaliation - Fearing to be categorized as a problem | *“Every time he beats me, he threatens that he will abandon me with the children and never come back if I report to anyone I cannot afford the finances to care for my children so when I hear that I cool and leave everything.”* (Brenda)  *"I told my brother-in-law about the abuse, but it made things worse. My husband attacked me that night, and now I only tell my mother."* (Sarah)  *"I chose to endure the abuse because this is my second relationship, and my previous partner was also abusive. I'm afraid that if I leave again, people will think I'm the problem."* (Medius) |
