# Supplementary material for: Intimate partner violence experience, support seeking and coping strategies among pregnant women in Southwestern Uganda
Source: PLOS Glob Public Health. 2026 Jan 5;6(1):e0002606. doi: 10.1371/journal.pgph.0002606 (PMC12768344; doi:10.1371/journal.pgph.0002606)
Supplement: Attachment — Submitted filename: Response to reviewers.docx [file pgph.0002606.s003.docx]

**Response to reviewers**

1. The background section now includes recent literature on IPV in pregnancy
2. To increase coherence, the first statement has been rearranged by combining the perspectives from Uganda, sub-Saharan Africa, and the world.
3. The entire manuscript has had extensive grammar editing.
4. Quotation marks have been added appropriately
5. Capitalization in the middle of the sentences has been avoided
6. Unnecessary bold font has been corrected
7. Coping strategies clarified
8. "The main objective stated in the background has been rephrased."
9. I added a theoretical framework

**Methods**

10. Goal to “achieve neutral findings” has been reconstructed for clarity
11. “Nominated sample” has been corrected
12. Line 118 has been revised to “2018 hospital database records of new and returning ANC cases
13. 118-122 edited and broken down into two sentences
14. Lines 126-127: Information has been provided regarding the saturation of data.
15. Line 128 revised to “in-depth”
16. Lines 130 -138 editing done, topics that were in the interview guide have been added
17. Lines 139- 140 Editing was done, and the field notes' purpose was added.
18. Line 154: I have added a reference for thematic data analysis.
19. The data was analyzed manually, without the use of any software for the analysis.
20. Line 158 Scripts revised to transcripts
21. Line 185, women who received support have been deleted since the section described methods
22. Lines 206-207 Have been separated into two sentences
23. Lines 209-213 clarification was put in line with Guba and Lincoln’s criteria

**Results**

24. The synthesis has been reworded and some quotes have been removed to shorten the results section.
25. Table 1: Variable and category columns have been collapsed
26. Theme one: Theme one: has been rephrased to various IPV experiences
27. Lines 236-239: Have been revised to distinguish between actions of the abuser versus actions of women experiencing IPV
28. Line 252 “added
29. Line 491 typo corrected and in general grammatical mistakes rectified
30. Lines 513 to 527 quotes reduced to three
31. Line 527 Saying yes to everything has been commented on and discussed

**Discussion**

32. Information regarding previous studies has been added in the discussion section
33. Clarification on countries where studies were done has been rectified
34. Line 663: “Women are never bread winners” has been deleted
35. 664: African tradition has been removed
