# Supplementary material for: Intimate partner violence experience, support seeking and coping strategies among pregnant women in Southwestern Uganda
Source: PLOS Glob Public Health. 2026 Jan 5;6(1):e0002606. doi: 10.1371/journal.pgph.0002606 (PMC12768344; doi:10.1371/journal.pgph.0002606)
Supplement: Attachment — Submitted filename: Response_to_Reviewers_auresp_2.docx [file pgph.0002606.s004.docx]

**Response to reviewer’s comments**

| # | Comment | Response | Page number/line number |
| --- | --- | --- | --- |
| 1 | This section needs expansion. Include: inclusion criteria for participants, how they were identified as having experienced IPV, where and by who they were approached to participate in the study. Where were interviews conducted?    Were women paid? | Thank you very much for the comments.  In methods section, the Inclusion criteria, how women were identified, where and who approached them have been added.  During the interview, each respondent received refreshments in the form of a soft drink and a snuck. No monetary incentives were offered. | Pg.6 -7  Ln. 172 -220  Pg. 8  Ln. 235-237 |
| 2 | Line 180 – consent waived for minors.  Should this refer to parental consent? | Thank you very much for the over sight  This meant that parental assent was waived because being pregnant at an age below 18 was considered as an “emancipated minor”. Therefore the authors were allowed by the Research Ethics committee to obtain consent from those pregnant women. | Pg. 8  Ln. 229-231 |
| 3 | Line 181 - suggest this should read “ identifying information” rather than “identity” | Thank you very much for the comment.  “identity” changed to “identifying information” | Pg. 8  Ln. 231 |
| 4 | If interpretive phenomenological analysis was used, some rationale for this choice and brief summary of the approach with references should be included. | Rationale for interpretive phenomenological analysis used has been added and a brief summary of the approach with references included. | Pg. 6  Ln. 154-160 |
| 5 | Rigor Line 196-197  These features guaranteed that data collection and content were consistent. It is suggested that to claim these measures provided a “guarantee” is an unwise.  Rather, I suggest you state the measures “ supported” consistency. | Thank you very much for the comment.  The term consistency has been used appropriately consistency. | Pg.9  Ln-249 |
| 6 | As a qualitative piece it does not yet add new material other than a description of the nature of abuse experienced by this group of women.  To establish the credibility of the findings, it is suggested that each theme is defined and articulated.  As part of this, and the analysis indicates the strength of the theme and any groups who this did not apply to.  As written in the current manuscript, each theme simply includes individual accounts to illustrate the theme.  These work as examples of a theme, but do not by themselves constitute complete analysis. | Thank you very much for the comments.  The whole result section has been revised and themes re-organized  Themes specifically indicating the drivers of IPV, coping strategies and barriers to support seeking have been added and re-organized in a meaningful manner.  A supplementary file indicating the revised themes as also been included in the manuscript | Pg. 9-15 |
| 7 | There is no Discussion section and the Limitations are not relevant for qualitative research | Thank you very much for the comments.  The discussion section has been over hauled and re-discussed in relation to key study findings.  The limitation section has been revised in relation to weaknesses of qualitative studies | Pg. 16 - 21 |
