# Supplementary material for: Intimate partner violence experience, support seeking and coping strategies among pregnant women in Southwestern Uganda
Source: PLOS Glob Public Health. 2026 Jan 5;6(1):e0002606. doi: 10.1371/journal.pgph.0002606 (PMC12768344; doi:10.1371/journal.pgph.0002606)
Supplement: Attachment — Submitted filename: Response_to_reviewers_auresp_3.docx [file pgph.0002606.s005.docx]

**Response to Reviewer’s comments**

| # | Comment | Response |  |
| --- | --- | --- | --- |
| 1. | **Reviewer #4**  Title: Please note that the title in the manuscript differs from the title in the electronic system. | Thank you very much for the comments.  Unfortunately it appears that one of the reviewers did not gain access to the current revised manuscript  The authors changed the original title since did not represent wholly all the study findings. Therefore the new title is:  *“Intimate Partner Violence Experience, support seeking and Coping Strategies among Pregnant Women in Southwestern Uganda* *”* | Pg. 1  Ln. 1-2 |
| 2. | **ABSTRACT** This also differs substantially between the manuscript and the electronic system and I was not sure which version was more recent. | The abstract in the electronic system was more recent, due to the fact that the previous reviewers requested authors to define and articulate each theme to indicate clearly the strength of the themes and this changed significantly the presentation of results and the abstract. The new abstract is:  *“Intimate Partner Violence (IPV) during pregnancy remains a global health challenge, and Uganda is no exception. This study aimed to explore pregnant women’s experiences of IPV, support seeking and coping strategies in Southwestern Uganda. Pregnant women who experienced IPV during pregnancy were selected for in-depth interviews. Data saturation was reached after 25 participants. Guided by the feminist theory, data was analyzed deductively and inductively using thematic analysis. Pregnant women described their IPV experiences which were categorized under physical (e.g., being stepped on the gravid abdomen), psychological (e.g., verbal abuse), sexual (e.g., forced uncomfortable sexual positions) and economic forms of violence (e.g., being denied money for transport and food). Pregnant women described the key drivers of IPV as intergenerational violence, helplessness, and unbalanced power dynamics and gender roles. Some pregnant women used active coping strategies to overcome the effects of IPV such as sharing their experiences of IPV with a confidant. Whereas other pregnant women adopted passive coping strategies such as keeping silent. Some pregnant women did not seek help after IPV experience due to lack of awareness, women’s caregiving perceived challenges, partner over dependence, a feeling of shame, guilt, and fear. These findings indicate that various stakeholders need to prioritize IPV screening, care, referral, and sensitization in health facilities and communities. In addition, rules and regulations that protect the rights of IPV survivors should be strengthened, and perpetrators held accountable for their actions ”.* | Pg.2  Ln. 32-54 |
|  | **Introduction** |  |  |
|  | The authors may wish to define intimate partner violence at the outset. | IPV has been defined at onset as:  “*Intimate partner violence (IPV), is defined as the actual or threatened physical, sexual, psychological, and emotional abuse by current or former partner (WHO, 2005)*.” | Pg.3  Ln.60-61 |
|  | The authors alternate between IPV and the full words and I suggest that IPV can be used throughout after the first definition of the acronym. | Thank you very much for the suggestion.  We have consistently used the IPV acronym after defining it for the first time. |  |
|  | I am confused about the prevalence of IPV being 27% at all of the global, regional and national levels. I would expect prevalence estimates at each of these levels would differ somewhat. | Thank you very much for the comment.  27% is the global life time prevalence of women aged 15 to 49. This has been corrected | Pg. 3  Ln. 62-63 |
|  | Line 57 - I understand what the authors mean by "IPV is a tradition" - that there is a long history of it going back generations - but the phrasing could potentially be interpreted as justifying IPV as a cultural practice so I suggest rephrasing this sentence. | The statement has been rephrased as:  “*Research indicates that IPV is a tradition running through generations in many communities in Uganda which further perpetuates IPV ”* | Pg.  Ln 72-73 |
|  | Line 60 - the prevalence is stated above to be 40% so it does not seem accurate that "most" women experience IPV. | The statement with the word ‘Most’ has been changed to “some of the women”. | Pg. 3  Ln.74 |
|  | The authors may wish to acknowledge that many of the coping strategies identified focus on the woman accommodating abuse rather than addressing male behavior. | Thank you very much for the comment, the following sentence has been added to the manuscript:  “ These coping mechanisms tend not to address male IPV behaviours but rather they encourage the woman to tolerate IPV” | Pg. 3  Ln. 118-120 |
|  | Line 98 - how does IPV screening effectively reduce depression? | The following statement has been added to the manuscript to the manuscript: “*Early screening allows healthcare providers to intervene with counseling and other support services with the aim of preventing mental health conditions such as depression (WHO, 2013).*” | Pg. 5  Ln.128-128 |
|  | Line 122 - would feminist theory say anything about pregnancy being a time of IPV vulnerability because the unborn represents a rival to the man in a patriarchal society? | Thank you very much  This has been added: *“Additionally, jealousy and possessiveness may contribute to IPV during pregnancy because the male partner may perceive the unborn child as a "rival," for the mother's love and attention”* | Pg.6  Ln. 154-156 |
|  | As a qualitative study it would help to explicitly state the research questions the authors set out to answer. | Thank you very much for the comment.  The research questions have been stated as follows:  How do pregnant women describe their IPV experience, help-seeking behaviors and coping strategies after experiencing IPV? | Pg. 7  Ln. 159-160 |
|  | **Methods** |  |  |
|  | Line 146 - please explain how saturation was defined and determined. | This has been explained as:” *The team revised the themes to ensure that they accurately described the respondents’ views and verified their accuracy through follow-up interviews until data saturation was reached. ”* | Pg. 9  Ln. 228- 229 |
|  | Line 155 - "the potential reasons behind it" sounds as though IPV is sometimes "reasonable" or justifiable. Perhaps this could be rephrased e.g "associated factors" or "contextual factors". | The term “Contextual factors” has been adopted | Pg. 8  Ln. 196 |
|  | Line 173 - the authors say earlier that they used an interpretive phenomenological approach but then say they used thematic analysis. Can they provide more detail about their analysis and how IPA came into it? | Thank you for the comment. Interpretive has been deleted since we used both deductive and inductive thematic analysis technique which may not necessarily align with interpretive phenomenology. | Pg. 7  Ln. 165 |
|  | Line 176 - what does "checked themselves" mean? | This was previously deleted |  |
|  | Line 178 - the term "significant" in relation to responses sounds rather quantitative. Could the authors clarify what they mean? | The term “Significant” has been changed to relevant | Pg. 8  Ln. 224 |
|  | Line 180 - "repetitive" sounds a bit critical. Recurring? | The term “Repetitive has been changed to *recurring* | Pg. 8  Ln 225 |
|  | Line 198 - participants aged 15-17 years are likely to have been the most vulnerable. Can the authors elaborate on the concept of emancipated minors and why they would not need to consent? | Thank you very much for the over sight  This meant that parental consent was waived because being pregnant at an age below 18 was considered as an “emancipated minor”. Therefore the authors were allowed by the Research Ethics committee to obtain consent from those pregnant women. | Pg. 8  Ln. 238-342 |
|  | Line 210 - again, "perfect" is a rather positivist term. Enhance interview skills? | “Enhance interview skills” has been adopted | Pg. 9  Ln. 254 |
|  | Line 212 - Data is plural of datum so should be written as "data were" rather than "was" in all cases. | The word "data were" has been used | Pg.9  Ln.256 |
|  | Line 215 - "for dependability the study to dispose of individual analyst predisposition" - this is unclear - I think it is about attaining consensus and confronting bias but this needs to be more clearly explained. | Thank you for the comment,  Dependability was previously revised as follows  *“A thorough description of the study approach also allowed for dependability which permits the study to be replicated.”* | Ln. 262-263 |
|  | Line 223 - I think a semi-colon is needed before "this contributed to appreciation..." | The whole sentence was revised |  |
|  | Line 226 - removing bias and producing objective results are quantitative rather than qualitative objectives. It would help to consistently frame the methods in qualitative terms of rigour and credibility rather than objective truth. | “removing bias and producing objective results” has been changed to:  remove any potential bias of individual investigators and increased rigor and trustworthiness in the research process; this produced subjective results | Ln. 268-269 |
|  | Line 228 - transferability is not something that one can "guarantee". Again I suggest less positivist language. | The term "guarantee" has been replaced with enhance | Pg. 10  Ln. 263 |
|  | There is no mention of assistance provided to women for the abuse they disclosed, or safety protocols to address risks identified during participation. | For this study, a professional counselor was enlisted to provide emotional support to women experiencing IPV | Pg. 9  Ln. 244-245 |
|  |  |  |  |
|  | **RESULTS** | The authors observed that one of the reviewers did not access the revised copy by then, but the whole result section had been revised and themes re-organized as requested by the first reviewers.  Themes specifically indicating: “*coercive behavior in intimate relationships, the drivers of IPV, coping strategies and barriers to support seeking”* were added and re-organized in a meaningful manner.  A supplementary file (S1 table) indicating the revised themes has also been included in the manuscript. All the comments made under the results section have been addressed. | Pg. 10 - 19 |
|  | Line 251 - this sentence has some formatting errors: a capital P in the middle of the sentence, a space before the comma. | This was already eliminated in the previous overhaul |  |
|  | Line 257 - the statement about feeling depressed is not supported by the quotations below. This (impact on mental health) is an important point so I suggest providing a supportive quotation. | Supportive quotation has been added | Pg.12  Ln.329-330 |
|  | The section on IPV is broken down into straightforward categories but some of the quotations implicate manipulative behaviour that shows signs of coercive control;  I wonder if the authors wish to acknowledge this. | Thank you very much for the comments.  On IPV section the straight forward categories have been eliminated and *“coercive behavior in intimate relationships ”* emerged as the new theme with the following categories:  “*physical coercion and violence, reproductive coercion, psychological violence, sexual coercion and financial control and manipulation”* | Pg.9  295-300 |
|  | Line 325 - "claiming to have no biological mothers" sounds like the women are not being believed. Could this be rephrased? | This was eliminated during re-analysis |  |
|  | The groupings of types of coping are rather simplistic. These could be less granular and more thematic, for example grouping all the ways that women adapt their own behaviour to accommodate abuse or all the ways members of the community inadvertently reinforce abuse. | Under the coping strategies theme, two categories were developed: 1) Active coping strategies and 2) Passive coping strategies | Pg. Ln.414-415 |
|  | Women in quotations are often laughing ruefully but humour isn't mentioned as a coping strategy. | The authors observed that participants would laugh most of the times because they felt shy at what they were saying so it this was not considered as a coping strategy |  |
|  | The fractured family unit, for example missing parents (I imagine, due to HIV, although this is not mentioned) is not mentioned.  This could be developed further within the intergenerational transmission theme which is currently very brief (and might also fit better as a sub-theme of experiences rather than a standalone theme). | During re-analysis intergenerational violence emerged from the drivers of IPV theme | Pg. 14  Ln. 363-349 |
|  | Overall the analysis could be developed to be more inductive and bring together more thematic understanding. | Re-analysis was done | Pg. 10-16 |
|  |  |  |  |
|  | DISCUSSION | The discussion section was over hauled previously and re-discussed in relation to key study findings. |  |
|  | Line 450 - there are some grammatical errors in this sentence - an apostrophe after midwives and capital A used for antenatal care. | Thank you very much for the comments.  This was a summary of findings at the beginning of the discussion and was all deleted | Pg.19 |
|  | Line 457 - these types of violence are reported worldwide, not only in LMICs. | There was study that was done in a low income community in USA that was being compared with the findings | Pg. 17  Ln: 514-516 |
|  | Line 470 - "prevents most women from working hard". This sounds rather judgmental and I would suggest a more nuanced discussion of economic gender dynamics. | This has been re-discussed | Pg.20-21  Ln 551-563 |
|  | Line 483 - New Zealand? | This was previously corrected | Pg. 20  Ln.554 |
|  | Line 491 - which proof is being alluded to here? | This eliminated during the re- discussion |  |
|  | Line 511 - while the authors are to be commended for discussing safe sexual positions freely as well as women's comfort and pleasure, the length of description feels excessive and does not obviously relate to the focus of the paper. | This has been re-discussed | Pg. 20  Ln. 537-549 |
|  | Line 535 - where was this study conducted? How can the authors reconcile their findings with those? | The study was conducted in Iran, we have compared their findings with our findings. | Pg. 22  Ln. 612- 615 |
|  | Line 539 - again, this section feels excessively long and it is not clearly linking the authors' findings to existing evidence. They seem to be describing best practice but I was unclear whether they were saying what should happen or what does happen in some places. | The sentence was deleted during re-discussion |  |
|  | Line 558 - the authors here again repeat their findings, which have already been stated at the start of the Discussion. | The results at the beginning of the discussion have been deleted | Pg. 19 |
|  | The comments about harms of spending time in church and lack of balance in life neglect the greater concern that religious coping treats violence as an intractable problem and also places the burden of coping on women while taking no action against perpetrators. | This was eliminated during the re-analysis and re-discussion |  |
|  | CONCLUSION Line 615 - "ignorant" sounds critical. Not informed? While reasonable recommendations, few of the conclusions follow from the paper's findings. | The conclusion section was revised after re-discussion | Pg. 25 |
|  | Recommending education on safe sexual positions presumes that male partners are unaware of risks to the woman and fetus whereas in this paper, unsafe positions were an example given of deliberate abuse. | Another recommendation has been reconstructed |  |
|  | Line 643 - qualitative research prioritizes lived experience and realities; "recall bias and inaccuracies" do not make sense as limitations of the methods used. | The limitation section was revised in relation to weaknesses of qualitative studies | Pg. 22  Ln.667-678 |
|  | Reviewer #5: |  |  |
|  | I wish to thank the authors for their immense work on generating evidence on such an important topic such as IPV, and more so among pregnant women. | Thank you very much for the comment |  |
|  | The authors have done a good job responding to all of the reviewer comments. However, they will need to work on language (punctuation and sentence length).  The best option would be to submit the work for thorough English language editing. Below are a few suggestions for revision: | Thank you very much for the comment  The paper has been copy edited by a professional editor and cover letter has been attached. |  |
|  | Page 4 line 114-117: the sentence is too long and not easy to understand. The first part makes sense, but the confusion starts with the second part and more confusing with the third part. Kindly review for conciseness. | The sentence has been revised and shortened | Pg.4-5  Ln.131-135 |
|  | Page 6 line 162-170: It would be great to let the reader know if this facility is public or private run. Also, an idea about its location or catchment area – does this serve high, low or mixed SES groups? | This information has been provided | Pg. 7  Ln. 174-177 |
|  | Page 7 line 195-198: The sentence is too long and needs to be broken into at least two for better understanding. It is not clear what the authors seek to communicate – the experience of the research team member? The persons who conducted the interviews? That steps were made to establish rapport ahead of interviews? | The sentence has been shortened and more clarification made | Ln. 239-244 |
|  | Page 7 lines 204-206: this contains 4 related short sentences which can be combined to form two sentences. For instance, “The study used pseudonyms instead of data identifiers to ensure anonymity” or “Data saturation was achieved by the 25th interview but we added two more interviews for confirmation.” | These sentences have been adopted: “The study used pseudonyms instead of data identifiers to ensure anonymity”  “Data saturation was achieved by the 25th interview but we added two more interviews for confirmation.” | Pg. 8  213-216 |
|  | Page 8 lines 229-231: The sentence ought to be revised to eliminate a technical fault. Consent is obtained from adults and assent obtained from minors. Thus, @....the need for assent by parents…” is technically unacceptable and must be corrected to “consent by parents” | This sentence has been revised | Pg.9  Ln. 238-242 |
|  | Page 9 lines 254-256: The sentence needs to be revised for clarity and conciseness Generally, the authors must use punctuation to make it easier to read some of the respondent quotations used. I do understand that these were verbatim from local dialect to English. However, non-native speakers will find it difficult to understand the translated versions without careful use of punctuations. | The statement has been revised to:  *“To promote a common interpretation of the results, each analyst kept a separate reflexive journal in which they documented any problems that might have affected the analysis”.*  Careful use of punctuations in quotations has been emphasized | Pg. 10  Ln. 265-266 |
|  | Page 14 lines 391-392: revise sentence for clarity. Perhaps insert “and” between IPV management and thought. | The whole sentence rephrased | Pg. 17  Ln.445-448 |
|  | Lines396-397: Revise sentence for clarity, The current use of “lack of staff and inadequate time” can be improved. Suggestion, “participants noted that the number of staff on duty often meant less time available for each client, making it difficult for them to routinely screen for IPV” | Thank you very much and this statement has been adopted:  “*participants noted that the limited number of staff on duty often meant less time available for each client, making it difficult for them to routinely screen for IPV”* | Pg. 17  Ln. 451-453 |
|  | Lines 398-399: might be better to start a whole new sentence with “some participants indicated….” | Thank you very much.  The whole new sentence has been started | Pg. 17  Ln. 454-555 |
|  | Page 17 lines 481-485: The need to empower women economically is a general concept and not limited to the pregnant women in this study. Please revise to eliminate “pregnant” and pregnancy is just transient. | “pregnant” has been deleted | Pg. 21  Ln. 558-560 |
|  | Same for sentence in 504-505. Also the last sentence is not complete and appears to be missing some part. | Pregnant has been deleted  The last sentence has been revised | Pg. 21  Ln. 582  Ln. 587 |
|  | Page 18 line 498-500: Please revise for clarity and conciseness | The sentence has been revised:  *“This study highlighted pregnant women’s feeling of helplessness as a driver of IPV. Women who lacked financial means to care for the children independently, had to remain in abusive relationships.”* | Pg. 21  Ln.576-578 |
