# Supplementary material for: Intimate partner violence experience, support seeking and coping strategies among pregnant women in Southwestern Uganda
Source: PLOS Glob Public Health. 2026 Jan 5;6(1):e0002606. doi: 10.1371/journal.pgph.0002606 (PMC12768344; doi:10.1371/journal.pgph.0002606)
Supplement: Attachment — Submitted filename: Rebuttal letter June.docx [file pgph.0002606.s006.docx]

**Response to reviewers’/ Editor’s comments**

| No. | Comment | Response | Page No. |
| --- | --- | --- | --- |
| 1. | The paper would benefit from editing,  providing clearer grouping among the themes and more interpretive analysis. There is some repetition which could be removed to make the paper more concise. | Thank you very much for the observation  The copy has been edited and regrouping in some themes has been done. To make the paper more concise repeated statements have been removed. |  |
|  | **ABSTRACT** |  | Pg. 2 |
| 2. | Coercion is used interchangeably with violence here but it is a subtype of violence and abuse. E.g. I would not call violation in pregnancy "reproductive coercion" - I would call it rape or sexual violence. | Thank you very much for the comment.  "reproductive coercion" has been changed to sexual violence  Interchangeable use of coercion with violence has been avoided throughout the paper | Ln 38-41 |
| 3. | Some phrases are made less clear by being truncated, e.g.  "women's caregiving perceived challenges" | Thank you for the observation  For clarity, the phrase "women's caregiving perceived challenges" has been changed to “Negative experiences with health care providers” | Ln 47-48 |
|  | **INTRODUCTION** |  | Pg. 3-6 |
| 4. | Line 68 "several women" seems a bit misleading given the high prevalence mentioned in the preceding sentence. | "several women" has been changed to “ a high proportion of women” | Pg. 3  Ln 63 |
| 5. | Line 72 "is a tradition" sounds a bit like cultural normalization. Alternative wording would be helpful. | "is a tradition" has been replaced by “recurring pattern” | Ln 68 |
| 6. | Line 73 "vice" is also an unusual term here. Break the cycle, perhaps? | "vice" has been replaced by “Cycle” | Pg. 3  Ln 69 |
| 7. | Line 74 "above all some of the women still believe..." | “women continue to be socialised to accept unequal rights and roles” | Pg. 3  Ln 70 |
| 8. | Line 76 "linked several factors" sounds like a causal interpretation. Perhaps "identified several risk factors" for IPV is more accurate. | "identified several risk factors" has been adopted | Pg. 3  Ln 72 |
| 9. | Line 107 alexithymia is unexpected here. Can the authors clarify why women would experience this due to IPV? | “Alexithymia” has been deleted |  |
| 10. | Line 107-112 care is needed when describing ways that women accommodate IPV by changing themselves to placate abusive partners. I suggest some adjustment to the wording here to clarify that studies have identified a range of ways that women may seek consolation, support or safety (rather than it sounding like these are all good ideas). | “Studies have identified a range of ways that women may use to seek consolation, support or safety” This adjustment was adopted for clarity. | Pg. 4  Ln 101-102 |
|  | Line 125-127 - do these points (8x ANC contacts and IPV screening) refer specifically to Uganda? | No, these points refer to women globally and a WHO reference has been included. | Pg.5  Ln 116-117 |
|  | Line 128 in what way does IPV screening "effectively reduce depression"? | The word “”effectively has been deleted . |  |
|  | **METHODS** |  |  |
|  | It would help to provide a little more contextual detail about the study site and region. | A little more contextual detail about the study site and region. have been added | Pg.7  Ln 155-161 |
|  | Line 165 - can the authors elaborate on what they mean by a phenomenological approach? | “Phenomenological approach” has been changed to Phenomenological design and explained.  [The term "phenomenological design" describes a qualitative research approach that aims to explore and comprehend individual’s actual experiences with a given event. In our study, this design was used to examine how pregnant women experience and interpret IPV, support-seeking, and coping strategies allowing us to capture the depth and meaning of their personal narratives]. | Pg. 7  Ln:152-154 |
|  | Line 180 - can the authors specify whether the WHO questionnaire was a translated version in Runyankore or in English? | The tool was translated into Runyankore, the predominant local language in the study area | Pg. 7  167-168 |
|  | Line 183 - what does "baccalaureate nurse" mean? Those with an undergraduate degree? | "baccalaureate nurse" has been deleted and replaced by: “Research assistants who had completed **a Bachelor of Science in Nursing”** | Pg. 7  Ln: 169-170 |
|  | Line 199 - please provide the interview guide as a supplementary file. | An interview guide has been provided as a supplementary file 2 |  |
|  | Line 214 - how was the point of saturation determined? | Data saturation was reached when no new information emerged and responses became repetitive, confirming existing patterns and indicating that further interviews would yield no additional insights. | Pg. 8  Ln:193-196 |
|  | Line 256 - please describe the process of participant validation. | This was conducted by sharing interview summaries and preliminary themes with respondents. Their feedback was used to confirm the accuracy of interpretations and refine the final analysis. |  |
|  | Line 270 - what do the authors mean by "this produced subjective results". | This produced subjective results where the findings were “based on personal opinions of the respondents” |  |
|  | **Results** |  |  |
|  | Line 276-7 - this first sentence is not required. | The sentence has been deleted | Pg. 11 |
|  | The level of detail provided in Table 1 seems likely to be identifiable.  Rather than listing individual participant demographics, I suggest reporting the mean age and gravidity across the sample and percentages for marital status, trimester, occupation and education. | Mean age and gravidity have been added and percentages have been put for marital status, trimester, occupation and education. | Pg.12 |
|  | Overall, the analysis is at quite a surface level and the themes seem to represent the topic guide questions more than the content from participants. For example, the first theme (Coercive behaviour in intimate relationships) groups together experiences of violence, women's self-blame, and mental health impacts - which ought perhaps to be sub-themes or perhaps the top-level themes could be more interpretive. | Re-analysis has been done.  “Coercive behavior in intimate relationships” has been changed to “Power and control dynamics in intimate relationships” | Pg. 12  Ln:275 |
|  | Similarly, "Drivers of IPV" includes content about women's hopelessness (e.g. line 379-380) and being trapped with no options for escape, which are rather different to drivers. | This has been revised | Pg. 14-15  Ln.332-369 |
|  | A possible structure for the analysis could be findings at the levels of the individual (man and woman), community, and wider society. At present the separation of these is not very easy to follow. | Thank you so much for the observation.  Though, based on the theory used in the study and the analysis we made, we could not structure the findings at the suggested levels |  |
|  | As I mention above, coercion is used interchangeably with violence and it should not be. E.g. Stepping on the gravid abdomen is not coercion, it is physical assault or violence. Being violated due to pregnancy is not reproductive coercion, it is rape/sexual assault/violence. | These have been corrected | Pg. 12  278-281 |
|  | Line 319 - this quote has already been presented above. | Quote was deleted |  |
|  | Line 355 - "gave birth to physical violence" - is this an intentional play on words? | The wording has been changed |  |
|  | Line 440 Barriers to seeking support - the structure of this sub-section would benefit from re-organisation. The authors summarise a lot of points in the first, dense, paragraph and then list three quotations one after the other. It would help to walk the reader through the different sub-themes. In this first paragraph the authors also do not highlight the point about community reinforcement of abuse - e.g. the points at lines 454 and 464 - these show deep community acceptance/normalization of IPV and active efforts by those who could support women to discourage their help-seeking. | This section has been re-organised and quotes following one another has been avoided.  Community reinforcement of abuse has been highlighted. | Pg. 18  Ln:392-442 |
|  | Line 480 - "abandoned their first violent relationship" - again sounds like an error by the woman - perhaps "fled a previous violent relationship"? | "fled a previous violent relationship" has been adopted | Pg. 17  Ln:424 |
|  | **DISCUSSION** |  |  |
|  | The Results, Discussion and Conclusions have some repetition that could be removed to make the paper more concise. | Lines 507-509; 683-684 and 686-687 have been deleted |  |
|  | Line 515 - when referencing other literature, the authors do not acknowledge substantial literature on perinatal IPV from other parts of sub-Saharan Africa, especially Ethiopia and South Africa. | More substantial literature from Ethiopia and South Africa has been acknowledged | Pg. 18  Ln:446-447 |
|  | I repeat my comment that "coercion" should not be used interchangeably with violence or abuse. | This has been rectified throughout. |  |
|  | Line 520 - While this sentence is uncontroversial, it does not respond directly to the findings of the study. | This has been replaced by another recommendation. | Pg. 18  Ln: 451-453 |
|  | Line 533 - a natural recommendation from this finding would surely be that men require targeted education to address the root causes of hostility towards pregnant women and the unborn and help them find alternative outlets for their emotions than violence. | “Men require targeted education to address the root causes of hostility towards pregnant women and the unborn and help them find alternative outlets for their emotions than violence”. This recommendation has been adopted | Pg. 18  Ln: 463-465 |
|  | Line 556 - the women expressed that men prevented them from accessing vocational opportunities which were available - so it seems that the recommendation should be about working with men around the acceptability of women's empowerment in addition to any interventions to offer skills and income generating activities. | “working with men around the acceptability of women's empowerment” has been included | Pg. 20  Ln: 484-485 |
|  | Line 566 I am not sure that the paper found that intergenerational violence was "one of the main" drivers. | “Main” has been deleted | Pg. 20  Ln: 493 |
|  | Line 575 - would the authors also advocate action at the community level? There have been high profile studies in Uganda (e.g. SASA!) which showed this to be effective. | Advocacy for action at the community level has been included | Pg.20  Ln:501-505 |
|  | Line 576 - I would have thought that helplessness is less of a driver and more of a consequence that then reinforces the persistence of IPV. The description in this paragraph seems to somewhat repeat the text above about economic dependence. | The whole paragraph has been deleted |  |
|  | Line 586 - again, while men's involvement in childcare is to be encouraged, I am not sure that this recommendation arises from the study's findings. | The whole of the statement and the corresponding recommendation were all deleted |  |
|  | Line 604 - it should be discussed here that the people women shared their problems with were often not supportive and sometimes actively encouraged them to tolerate abuse - and link this to the many studies finding the same in sub-Saharan Africa and elsewhere. | This has been done | Pg. 22  Ln: 528-533 |
|  | Line 605 - sharing with church leaders is not mentioned in the Results. | A quote has been added in the body of results since it was an oversight. | Pg. 16  Ln: 377-380 |
|  | Line 613 - the sample who took part in this study were, by definition, known in ANC to be experiencing IPV. So this sub-group of women would not represent the group of women who do not disclose abuse. So I am not sure that the findings of this study do contradict the Iranian study, necessarily - there may be many women out there who the ANC providers do not know about. | “Findings contradict the Iranian study”: This has been completely eliminated. |  |
|  | Line 623 "passive coping strategies" - I wonder if a better phrase is "accommodating" abuse? Women adapt their behaviour as a survival tactic to avoid provoking a volatile partner's anger. | Thank you for the observation  "Accommodating abuse” has been accepted and added. | Pg.22  Ln:539-542 |
|  | Line 648 - there are other reasons midwives may not inquire, including their own patriarchal norms and personal experiences of IPV victimisation. | “Reasons why midwives may not inquire about IPV experience in pregnancy include patriarchal norms, personal experiences”. This has been added with appropriate citation | Pg. 23  Ln:566-568 |
|  | Line 653 - it would be helpful in the methods to clearly state what (if any) training on IPV is currently provided to ANC providers in the study context. | The ANC providers in the study environment have not received any kind of IPV training. On the other hand, the ANC card now includes a gender-based violence (GBV) screening item. | Pg. 7  Ln:163-165 |
|  | **LIMITATIONS** |  |  |
|  | Line 677 - qualitative research does not set out to be generalizable to other contexts so this is not a limitation of the design. | Thank you very much for the observation. The statement has been eliminated. |  |
|  | However, the authors have not commented on the data being 6 years old and the many contextual changes that will have taken place in that time. It would help to briefly explain in the methods why the analysis has been conducted several years later and to acknowledge contextual differences between now and 2019 in the Limitations section. | Data being 6 years older has been commented on, in the methods section.  Contextual differences between now and 2019 have been acknowledged in the Limitations section. | Pg. 9  Ln: 210-214 |
|  | A strength may be that the study is conducted in a region of Uganda less well-represented in existing research - is that the case? | Thank you very much for the contribution!  *“The study's strength is that it was carried out in an area of Uganda that hasn't been as well-represented in previous studies”.*  This has been accepted and added | Pg. 25  Ln: 594-598 |
|  | **MINOR POINTS** |  |  |
|  | Data is plural of datum so should be treated as plural throughout e.g. line 37 should say "data were..." | Thank you very much for the observation.  "Data were..." This has been corrected. | Pg. 2  Ln: 37 |
|  | I suggest stating "years" after all age ranges e.g. line 181 | The word “years” has been added after all age ranges | Pg. 7  Ln: 170-171 |
|  | Line 290, 292 - Themes are created interpretively by the analysers - so it is questionable whether the term "emerge" is applicable. The authors might want to use other terms e.g. "we identified x themes". | Thank you very much for the input  "We identified x themes" This term has been accepted and applied | Pg. 12  Ln:270 |
|  | The term "accurate" is used several times in the Methods but this is a rather a positivist term. | The term "accurate" has been replaced with “authenticity” |  |
|  | A careful grammar edit is needed for the small number of errors e.g. Line 570 - "what they have ever seen their parents' doing". | “Children who witness experiences of IPV may be more likely to replicate such behaviors in their own adult relationships” | Pg. 20  Ln: 497- 498 |
