# Supplementary material for: Intimate partner violence experience, support seeking and coping strategies among pregnant women in Southwestern Uganda
Source: PLOS Glob Public Health. 2026 Jan 5;6(1):e0002606. doi: 10.1371/journal.pgph.0002606 (PMC12768344; doi:10.1371/journal.pgph.0002606)
Supplement: Attachment — Submitted filename: Rebuttal letter.docx [file pgph.0002606.s007.docx]

**Response to reviewers’ comments**

| No. | Comment | Response | Page/line number |
| --- | --- | --- | --- |
| 1. | Line 231 can the purposive sampling techniques be briefly described. Which participant characteristics were being sought? | This has been rectified; details have been added to clarify. | P. 8  175-177 |
| 2. | Line 901 the authors have replaced the sentence suggesting that lack of generalizability is a limitation with an almost identical sentence. As I said, previously, this is not a valid critique of qualitative methods as this is not their purpose. | This sentence was deleted in the previous submission during proofreading of the final copy, and therefore was not in tracked changes. |  |
| 3. | **ABSTRACT** Line 46: I suggest "their households" or "the household". | We have used “their households.” | P. 2  Line 42-43 |
| 4. | Line 47: Differences in traditional roles and norms between what and what? Between the man and woman? Between his upbringing and modern Ugandan women's lives? | We have used “…differences in traditional roles and norms between men and women.” | P. 2  Lines 43 & 44 |
|  | INTRODUCTION Line 71: "years" is still not used uniformly following age ranges. | Thank you for this observation. We have added “years” to clarify. | P. 3  Line 64-65 |
|  | Line 125: watching movies is, I assume, a coping strategy available only to women with a certain degree of affluence? Is it an appropriate example for this study sample? | “watching movies on television ”  This coping strategy was retained and edited a little as the study was conducted in a city setting where it is common for all households to possess small and cheap television sets that enable them to access movies at home. | P.3  Line 101-102 |
|  | Line 156 prevent and treat mental health conditions? | We have used “…allows maternal mental health conditions to be prevented and treated.” | P. 5  Lines 118–119 |
|  | Line 162 I suggest deleting "However". | “However” has been deleted. | P. 5  Line 124 |
|  | METHODS Line 280 I would suggest "would be unlikely to provide..." rather than "would not provide". | We have used “…would be unlikely to provide...” | P. 8  Line 196 |
|  | Line 347 I suggest deleting "objective" as this term is not in keeping with the qualitative approach used. | We have deleted “objective.” | P. 11  Line 253 |
|  | RESULTS Table 2: this is much more confidential, thank you. Please however add units where appropriate, e.g. "Mean age (years)" and "Mean gravidity (number of pregnancies". | We added “Mean age (years)” and “Mean gravidity (number of pregnancies)” to clarify. | Page 12 |
|  | Line 369: The authors have deleted "emerged" from the text but not the heading. | We replaced “emerged”  with “identified ”in the subheading. | P. 12  Line 270 |
|  | Line 533 I suggest deleting "were" in "some were refrained from earning..." | We deleted “were.”  Thank you for the observation. | P. 15  Line 361 |
|  | DISCUSSION Line 828 Perhaps additional "intervention" research rather than "quantitative" research? | We have used “intervention.” | P. 22  Line 543 |
|  | Line 904-5 "less well-represented subject" - perhaps "under-represented" is clearer? | We have used “under-represented.”  Thank you for the constructive comments throughout this journey. | P. 25  Line 588 |
|  | GENERAL A grammar check is needed. Issues include the following: The tense - past tense is used often in the Introduction where present tense would be more natural. | A grammar check has been performed for the whole document. |  |
|  | The need for clarity following corrections, e.g. "making it makes it difficult" (line 79). | We deleted “makes it”  in the previous copy during proofreading. |  |
|  | Lack of agreement: - "woman's age, women's decision making ability" - I suggest agreement i.e. both being "woman" or "women" (line 86) | This has been corrected (i.e., “women’s age”). | P.3 Line 73 |
|  | - "Uganda, Tanzania, Ethiopia and in New Jersey" - I suggest replacing New Jersey with the United States as all the other examples are countries (line 824). | “US” has been used in place of New Jersey. | P. 20  Line 463 |
|  | - "data was gathered" (line 896) should be "data were..." Punctuation (e.g. "a victims' isolation" (line 111). | “Data were” was corrected in the previous version although it was not captured in the tracked changes.  We have deleted “a” so it now reads “victims’ isolation.” | P. 9  Line 200  Line 93 |
|  | Fragments in the text (e.g. "On the other hand," line 132, "Pregnant women..., line 313-4 - this sentence is a grammatical) | This has been addressed | P. 5 |
|  | Use of articles e.g. "Although the Uganda's..." (line 159). Inappropriate use or lack of capitals (e.g. "some IPV victims", line 134; "conducted In-depth interviews", line 257). | This has been addressed. Thank you very much |  |
