# Supplementary material for: Intimate partner violence experience, support seeking and coping strategies among pregnant women in Southwestern Uganda
Source: PLOS Glob Public Health. 2026 Jan 5;6(1):e0002606. doi: 10.1371/journal.pgph.0002606 (PMC12768344; doi:10.1371/journal.pgph.0002606)
Supplement: Attachment — Submitted filename: Response to Reviewers.doc [file pgph.0002606.s008.doc]

| **Response to review comments** | | | |
| --- | --- | --- | --- |
| **#** | **Comment** | **Response** | **Page or line#** |
| 1 | Journal Requirements:  1. We would like to request copyediting.  If the reviewer comments include a recommendation to cite specific previously published works, please review and evaluate these publications to determine whether they are relevant and should be cited. There is no requirement to cite these works unless the editor has indicated otherwise.  Please review your reference list to ensure that it is complete and correct. If you have cited papers that have been retracted, please include the rationale for doing so in the manuscript text, or remove these references and replace them with relevant current references. Any changes to the reference list should be mentioned in the rebuttal letter that accompanies your revised manuscript. If you need to cite a retracted article, indicate the article’s retracted status in the References list and also include a citation and full reference for the retraction notice. | Thank you very much for the comments.  The manuscript has been copy edited by a professional English editor (Ms. Audrey Holmes)  All relevant articles have been cited  The reference list has been reviewed, and it is complete. No retracted article is cited |  |
|  | **REVIEWER # 4** |  |  |
|  | Reviewer #4: Thanks you for your comprehensive attention to their editorial comments and has accepted your paper for publication without further peer review. Only very minor revisions addressing grammar and syntax are needed from this reviewer. | Thank you very much for the compliment and time. The minor comments have been addressed. |  |
|  | Reviewer #4: Thank you to the authors for their comprehensive edits to this manuscript. I noted only some very minor points which do not require peer review once addressed. Line references refer to the tracked changes version. | Thank you very much for the compliment and time. The minor comments have been addressed. |  |
|  | Line 70 aged 15 years "and over"? | Thank you very much for the suggestions. We have changed the statement from 15-years to “15 years "and over | Ln65 |
|  | Line 151 it looks like a word or two is needed at the start of this sentence. | Thank you very much for the suggestions. The phrase “The theory” has replaced “it” at the beginning of the sentence | LN 139 |
|  | Line 171 I suggest changing 1 back to "one" month | Thank you very much for the suggestions. 1 has been written in words as “one” | Ln 157 |
|  | Line 299 would "sub-themes" be preferable to "categories"? | Thank you very much for the suggestions. We have inserted the word “subthemes” in the parentheses of the word categories. Since we use categories in the tables, we may not change them here for purposes of clarity | LN 208 |
|  | Line 482 I believe "chose" (past tense) would be better than "choose". | Thank you very much for the suggestions. The word “choose” has been changed to “chose” | LN 436 |
|  | Line 580 "keep and calm" should I think be "and keep calm". | Thank you very much for the suggestions. The sentence has been edited from "keep and calm" to “tolerate abuse and keep calm” | LN 527 |
|  | Line 599 "further implementation research..." As this sentence is about exploring impacts on women I suggest deleting "implementation" as this would only have relevance for an intervention focus | Thank you very much for the suggestions. The term “implementation” has been deleted | LN 543 |
|  | REVIEWER #6 |  |  |
|  | **Abstract**   - The abstract is overall well written. However, it should briefly explain **how participants were selected** for in-depth interviews (e.g., snowball sampling, convenience sampling, etc.). | Thank you for the comment, we have inserted the sampling technique and the sentence now reads as below:  “Pregnant women who had experienced IPV during pregnancy were purposively selected and completed in-depth interviews, with data saturation reached after 25 participants” | Pg  Ln 35 -36 |
|  | - The definition of **reproductive coercion** is vague. The authors should clarify whether they intend to treat coercion as a distinct form of IPV or as a mechanism through which IPV occurs (e.g., being coerced into sexual intercourse). | Thank you for the comment,  Coercion was considered as a means through which IPV occurs. In agreement with previous reviewers the word “coercion” was basically eliminated from the manuscript.  However we used the WHO categorization of IPV by WHO guidelines (WHO, 2005). |  |
|  | - If **thematic analysis** was used, findings in the abstract should be organized around the identified themes. For clarity, indicate **how many themes emerged** and mention them directly, rather than presenting the findings in a generic way. | Thank you very much for the comment. Based on the current guidelines on presentation of findings can be by themes or presentation as a storyline and we opted for the latter. However themes are presented in the main result section of this manuscript. |  |
|  | **Introduction**   - **Line 46:** Clarify whether the WHO global estimate of 27% IPV prevalence refers specifically to women aged **15 years only** or **15 years and above**, as this has implications for the study’s sample. | Thank you very much for the suggestions.  We have changed the statement from 15-years to “15 years "and over. And our sample population was within the age of 15 to 49 years according to the WHO guidelines regarding the age group at risk of IPV. | Pg. 3  Ln 71 |
|  | - **Line 75:** The sentence on cultural norms influencing IPV feels incomplete, especially since the next section moves into forms of IPV. For better flow, define IPV first, then introduce the cultural context. | Thank you very much for the comment.  We have re-organized the paragraph on the types of IPV and placed cultural norms after the definition of IPV.  We have added some examples of cultural norms that influence IPV. | PG 3  Ln 61-67 |
|  | - **Line 83:** The transition to IPV effects on pregnancy is abrupt. Strengthen the link from earlier discussion to emphasize why pregnancy is central to this study. | Thank you for the comment. We have strengthened the transition by adding reasons why women during pregnancy are at increased risk to IPV  The following statement has been added:  “Pregnant women are more likely to experience IPV, due to their increased vulnerability, dependency, and stressful life transition. This is because pregnancy can increase a woman's physical and emotional dependence on her partner due to financial demands, physical restrictions, and the need for support which may embolden abusive partners to assert control. Anxiety about money, raising a child, and shifting roles in relationships can also be triggered by pregnancy”. | Pg. 4  Ln 84-89 |
|  | - **Lines 95–112:** This section summarizes previous literature on coping strategies, but it is unclear what the **novel contribution** of this study is. Clarify whether earlier studies were conducted in other regions, relied on quantitative methods, or addressed different populations. Explicitly state the **gap** your study is addressing. | Thank you for the comment.  We have emphasized the following study gap:  “Most importantly”, quantitative research methods have been used to identify the majority of these strategies among women in general.” | Pg. 4  Ln 121-122 |
|  | - **Line 114–115:** The mention of developing interventions seems misplaced in the introduction. This would be better suited for the recommendations or implications section. | It was important to include this because we needed to show the reader the significance of conducting the study upfront. However recommendations are still added in the discussion based on the study findings. | Pg 6  Ln 131-138 |
|  | - **Lines 125–127:** Prior studies cited here examined prevalence and associated factors, which differs from the focus on coping strategies in the present study. Clarify this distinction and position your work as the for example **first to qualitatively explore IPV experiences and coping strategies during pregnancy in this context**???...only if it is | Thank you for your comment.  Although this comment is unclear, in line 131 -138, we mention our gap in line with the topic and we also provide other literature gaps in relation to IPV in pregnancy as well we state our objective in line with your recommendation. |  |
|  | **Study Population and Recruitment**   - The manuscript notes that the study is part of a larger project with 283 pregnant women. Please clarify:   - Was the qualitative subsample of 25 participants drawn from this larger cohort?   - What were the aims of the larger study, and how does this qualitative component fit within it? Without this context, readers may assume that all 283 participants were part of the qualitative study. - Consider separating the description of the **larger study** from the **qualitative component** to avoid confusion. - Clarify whether 283 participants were actually enrolled within one month, as this appears unlikely and overly ambitious. | Thank you very much for the comments.  The primary study of 283 which aimed to establish the prevalence of IPV disclosure and associated factors among pregnant women was used to draw 25 participants for this study from 199 who had experienced IPV among the 283 pregnant women.  This has been clarified in the manuscript as.  “The lead researcher (midwife) then used purposive sampling techniques to select 25 participants for in-depth interviews among a total of 199 women who had experienced IPV”  We have separated the two and started the qualitative component in a new line. Though we cannot completely eliminate the larger study because the current study sample size depended on the previous study.  We have clarified that we collected data from 25 pregnant women in one month. | Pg. 8  LN 176-188 |
|  | - Clarify how **consent was obtained from minors (ages 15–17)**. By law, parental consent is generally required unless participants were recognized as emancipated minors and the ethics committee formally waived guardian consent. Please specify which applied. - **Lines 224–226:** The explanation of consent for participants aged 15–17 is ambiguous. Confirm explicitly whether:   - parental consent was not required because participants were emancipated minors, or   - the ethics committee waived the requirement for parental consent | Pregnant women who were aged 15-17 years were considered as emancipated minors and this was clearly stated under ethical consideration.  Parental consent was not required since pregnant women aged 15-17 were considered as emancipated minors. | Pg 10  Ln 234-238 |
|  | **Data Analysis**   - **Line 208–209:** The phrase “categories were deductively related to the aims of the study” is confusing. Please clarify how categories were generated and linked to the study aims. | Thank you for the comment.  We familiarized ourselves with the transcripts, developed codes from the transcripts and later on categories were developed from codes. Since the study aims acted as themes, categories were linked to them. All this information is clarified in the previous lines before 208-209. | Pg 9-10  Ln 215-220 |
|  | - **Lines 212–214:** Specify what is meant by “several years” of experience among the researchers. Providing an exact range would be clearer. | The comment given of several years referred to the period when data was analysed after collection and manuscript submitted to the journal.  It does not refer to the experience of the researchers  Data was analysed after 3years of data collection and this has been added to the manuscript | Pg. 10  Ln 224-225 |
|  | **Main Themes**   - The manuscript states that both **deductive and inductive analysis** were used, but the themes appear to have been largely guided by the study aims (deductive). If inductive analysis was also applied, more participant-driven themes should be evident. - Themes such as *Drivers of IPV* read more as broad categories than actual emergent themes. Within these, themes like *power dynamics* or *intergenerational violence* could be identified. - There is overlap between the first two themes, suggesting they might be merged or restructured. - Similarly, *Coping Strategies* seems more appropriate as a **domain or subsection**, from which themes (e.g., faith-based coping, seeking family support, and silence/avoidance) would emerge. | Thank you very much for the suggestions  We agree with you that most of the themes were deductively developed and a few themes were participants driven, however we cannot re-analyse and re-organize the themes since we have done this several times under the guidance of previous reviewers. Since every reviewer has his own perspective this implies that we will have to keep changing the themes each time we obtain a new reviewer and this will distort the whole discussion and conclusion. Therefore we humbly request we maintain the current state of the themes. |  |
|  | **Strengths and Limitations**   - The current discussion presents some limitations as strengths. For example, findings from one clinic with 25 participants cannot be generalized to other populations, particularly women with different socioeconomic or educational backgrounds. - The claim that saturation was achieved with 25 participants should be reconsidered. Qualitative research shows saturation can occur with as few as 6–12 participants; thus, sample size alone is not a strength. - Recruiting participants from the same clinic may also have introduced bias, as participants might share information with one another. - Given that themes appear to have been predefined, there is also the potential for **researcher bias** in coding. - Please include a section on the **implications** of the findings for practice, policy, or future research | Thank you for the comments.  We have added the two limitations in the discussion.  The claim that saturation was achieved with 25 participants has been reconsidered and removed as a strength.  The study having been conducted in one clinic may have introduced bias. In addition the fact that most themes were deductively linked to the study aim may have introduced researcher bias in coding.  The implications have been incorporated in the discussion in line with plosglobal public health guidelines |  |
|  | Reviewer #6: Requests minor revisions addressing methodological rigor and structural clarity. Key issues include: clarifying the relationship between the 25-participant qualitative study and the larger 283-participant project;  explaining participant selection methods and consent procedures for minors;  strengthening the introduction by better articulating the research gap and novel contribution;  restructuring themes to reflect genuinely emergent findings rather than predetermined categories;  addressing overlapping themes and potential researcher bias; reframing stated "strengths" as actual limitations (particularly regarding generalizability from a single clinic);  and adding missing sections on research implications. | Thank you very much  We have carefully responded to all these in the current manuscript. |  |
